# Supplementary material for: Graph Neural Networks for Charged Particle Tracking on FPGAs
Source: Front Big Data. 2022 Mar 23;5:828666. doi: 10.3389/fdata.2022.828666 (PMC8984615; doi:10.3389/fdata.2022.828666)
Supplement: Supplementary file 1 [file Data_Sheet_1.PDF]

Supplementary Material

1 ALTERNATIVE GRAPH CONSTRUCTION

In this supplementary material, we investigate an alternative graph construction to show how the algorithm may be applied in different particle tracking settings, specifically for $p_{\mathrm{T}} > 1\,\mathrm{GeV}$ graphs. The graph construction restrictions are $z_0 < 350\,\mathrm{mm}$ and $\Delta\phi/\Delta r < 0.0007$ and $p_{\mathrm{T}}^{\mathrm{min}} = 1\,\mathrm{GeV}$. Each event is divided into 8 ϕ sectors, and 8 η sectors. Example graphs for 2 sectors in one event are shown in Fig. S1.

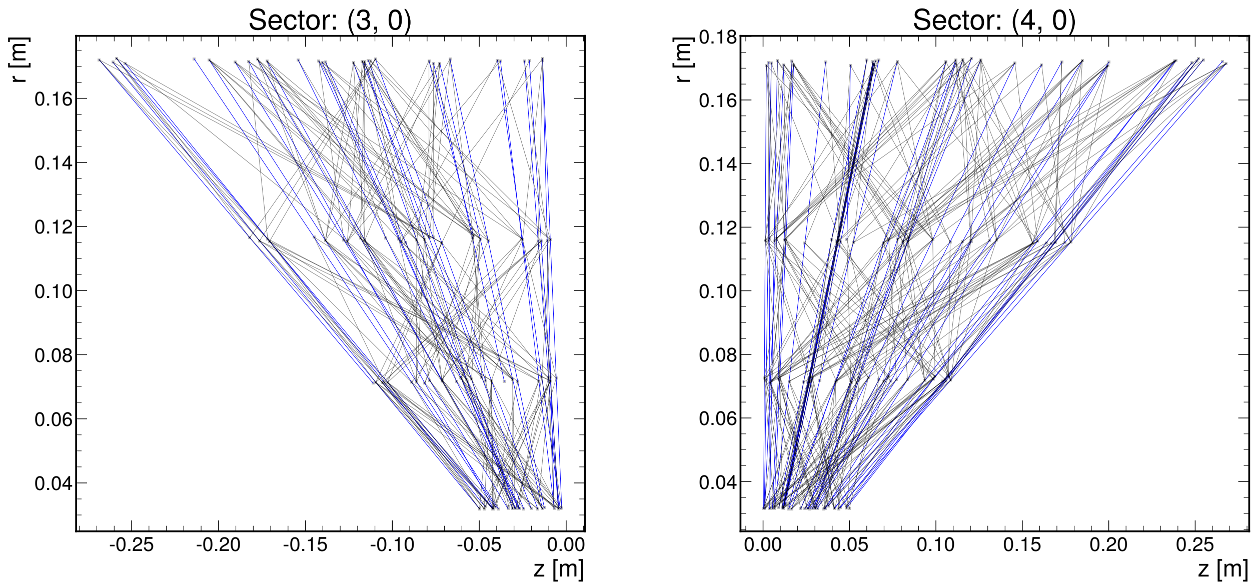

Figure S1. Example graphs showing 2 of the sectors for one event with $p_{\mathrm{T}}^{\mathrm{min}} = 1\,\mathrm{GeV}$, $z_0 < 350\,\mathrm{mm}$, $\Delta\phi/\Delta r < 0.0007$, 8 ϕ sectors, and 8 η sectors. True track segments are denoted by blue edges, while false track segments are denoted by gray.

The efficiency and purity of the graph construction method are shown for different choices for the number of ϕ and η sectors in Fig. S2 based on 50 events in `train_1`. In particular for 8 phi sectors and 8 η sectors, the graphs retain an efficiency of 97% and a purity of 50%.

Figure S3 shows the 95th percentile for the number of nodes and edges in each sector depending on the number of sectors chosen. For example, the average 95th percentile graph size for 8 ϕ sectors and 8 η sectors is 162 nodes and 326 edges for this graph construction. However, we note the distribution of nodes and edges depends on the particular $|\eta|$ range of the sector.

Figure S4 shows the AUC values for the 1 GeV graphs as a function of the total bit precision, where half of the available bits are used for the integer part and the other half are used for the fractional part. Different from the 2 GeV task, we see that with 16 total bits, we reproduce the 32-bit floating point model when applying the `ap_fixed<X,X/2>` PTQ scheme.

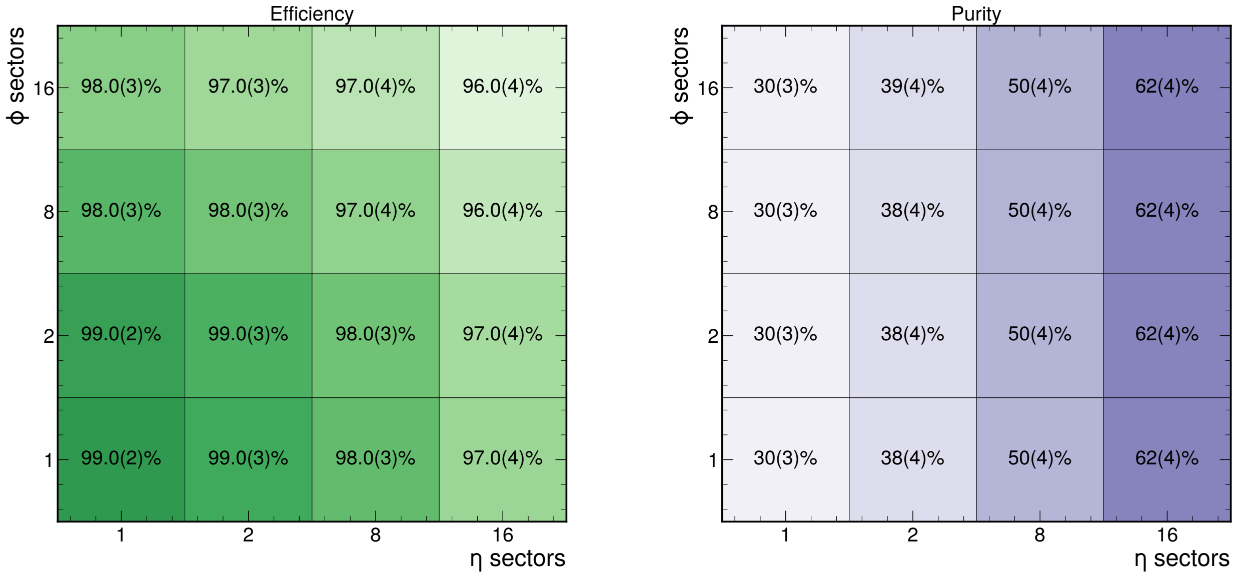

Figure S2. Efficiency (left) and purity (purity) of the 1 GeV hitgraphs studied for different numbers of η and ϕ sectors based on 50 events in `train_1`.

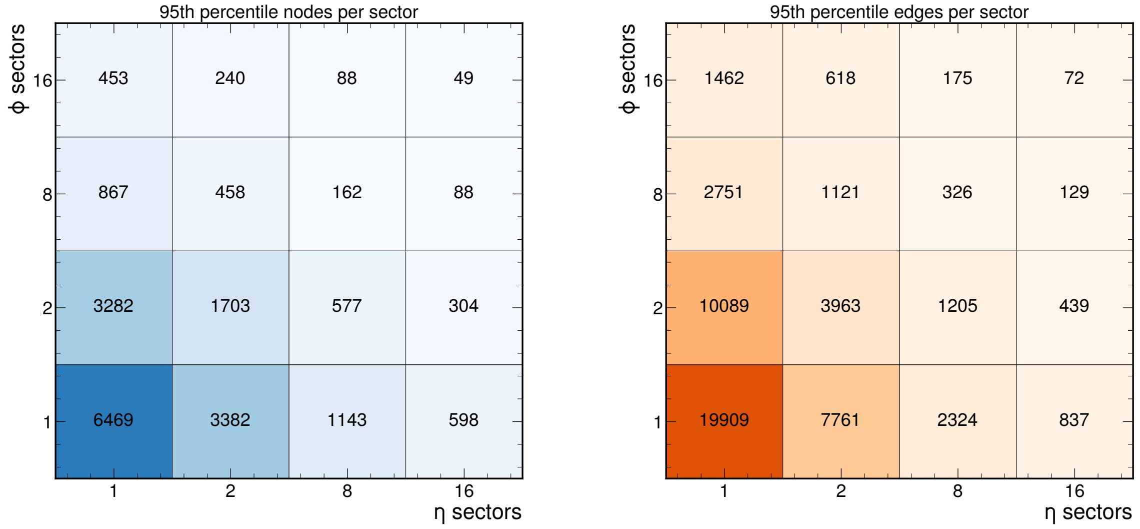

Figure S3. 95th percentile of the number of nodes and edges in each sector for the 1 GeV graphs as a function of the number of η and ϕ sectors, based on 50 events in `train_1`.

2 QUANTIZATION-AWARE TRAINING

Additional interaction network models were trained at different bit widths using the BREVITAS library to illustrate benefits of QAT. BREVITAS uses a scaled integer quantization scheme compared to the fixed-point precision scheme of `hls4ml`, however we expect the hardware resources and timing to be comparable for the same number of total bits. BREVITAS implements scaled integer quantization by assigning a zero

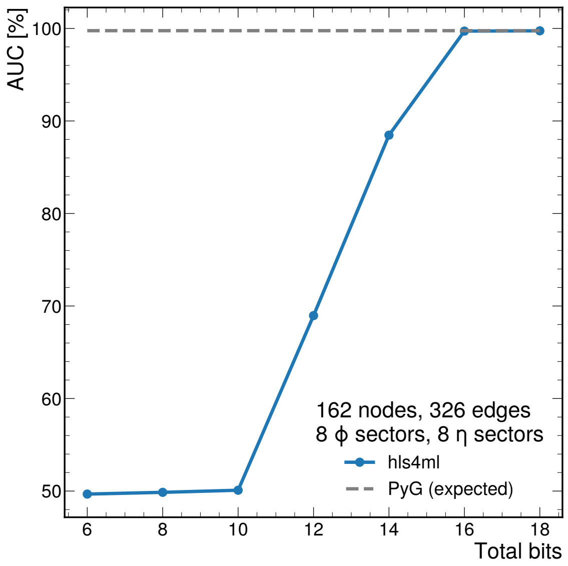

Figure S4. AUC values as a function of the total bit width `X` when using `ap_fixed<X,X/2>` with sectorized 1 GeV input graphs truncated at 162 nodes, 326 edges, corresponding to the 95% percentile graph size. The performance is evaluated with 1000 graphs from `train_2`. With precision greater than `ap_fixed<16,8>`, the AUC closely approximates the full floating point model.

point and scale factor for all of the inputs and activations. From there, the inputs and activations can be shifted and scaled to fit within the integer range defined by the bit width of the quantization. In the case that an input or activation exceeds the minimum or maximum value of the scaled integer range, the value is clamped at the boundary. As illustrated in the main text, the network retains the full performance even down to 7 total bits. PTQ will inherently reduce accuracy due to the loss of information that occurs when converting from the floating-point representation to the fixed-point or scaled integer representation. QAT allows for optimization while taking the loss of precision into account, allowing the network to train around the loss in precision and maintain accuracy. For this study, QAT models with bit widths from 2 to 18 were trained using the same training set (`train_1`) and evaluated on the same testing set (`train_2`) as the PTQ models to generate ROC curves and calculate the AUC at different bit widths.
